# Non-Invasive Breast Cancer Diagnosis through Electrochemical Biosensing at Different Molecular Levels

**DOI:** 10.3390/s17091993

**Published:** 2017-08-31

**Authors:** Susana Campuzano, María Pedrero, José Manuel Pingarrón

**Affiliations:** Departamento de Química Analítica, Facultad de CC. Químicas, Universidad Complutense de Madrid, E-28040 Madrid, Spain; mpedrero@quim.ucm.es

**Keywords:** breast cancer, circulating biomarkers, nanomaterials, electrochemical affinity biosensors

## Abstract

The rapid and accurate determination of specific circulating biomarkers at different molecular levels with non- or minimally invasive methods constitutes a major challenge to improve the breast cancer outcomes and life quality of patients. In this field, electrochemical biosensors have demonstrated to be promising alternatives against more complex conventional strategies to perform fast, accurate and on-site determination of circulating biomarkers at low concentrations in minimally treated body fluids. In this article, after discussing briefly the relevance and current challenges associated with the determination of breast cancer circulating biomarkers, an updated overview of the electrochemical affinity biosensing strategies emerged in the last 5 years for this purpose is provided highlighting the great potentiality of these methodologies. After critically discussing the most interesting features of the electrochemical strategies reported so far for the single or multiplexed determination of such biomarkers with demonstrated applicability in liquid biopsy analysis, existing challenges still to be addressed and future directions in this field will be pointed out.

## 1. Introduction

Breast cancer is one of the three most common invasive cancers in females and one of the leading causes of cancer mortality among women worldwide. Since distant metastases are regarded as the major reason of death, early diagnosis becomes vital for improving this cancer type survival rate [1,2].

Traditional breast cancer diagnostic tools include clinical and physical examinations, histopathology, imaging mammography, and ultrasound magnetic resonance imaging. Insufficient clinical and physical examinations drive patients into mammography or ultrasound studies. Ultrasound is a non-invasive and safe tool, but since it is unable to screen many of the cancers it cannot replace mammograms, especially in women above 40. Mammography has limited sensitivity, a high rate of false positive results and may also lead to an accumulated exposure to radiation, which is considered an additional risk factor. Histopathology is an invasive approach to examining cancerous tissues once the disease is installed. All these limitations demand urgently the development of non-invasive, simple and low risk methods suitable for point-of-care (POC) screening/diagnosis of breast cancer. Undoubtedly, the international oncology community is in active pursuit of non-invasive methods adequate to be implemented in clinical routines for the diagnosis of breast cancer patients. Currently, strong efforts are being developed to monitor specific bodily fluid biomarkers for early and minimally invasive detection of this type of cancer [3].

In this sense, electrochemical biosensors, combining selective biochemical recognition with the high sensitivity assumed for electrochemical detection, show particularly attractive merits such as disposability, portability, simplicity, low cost, small size, rapidity of response, ease of use, possibility of reading small sample volumes directly over a wide range of concentrations and compatibility with POC testing [3,4,5,6]. 

The need for ultrasensitive determination of cancer biomarkers of different natures in minimally invasive samples has led to the coupling of the benefits of electrochemical biosensing with those of nanotechnology to generate a new class of low cost, robust, reliable, easy-to-use, and ultra-sensitive diagnostics tools. Nanomaterials with unique features, such as noble metal and metal oxide nanoparticles (NPs), nanochains (NCs) and nanospheres, and integrated nanostructures including graphene or reduced graphene oxide (rGO), or composed of metallic NPs or oxides and multiwalled carbon nanotubes (MWCNTs), do provide biosensors for the determination of circulating breast cancer biomarkers with high sensitivity and precision and low detection limits (LODs) [7].

## 2. Circulating Breast Cancer Biomarkers 

A biomarker is defined as “a biological molecule found in blood, other body fluids, or tissues that is a sign of a normal or abnormal process or of a condition or disease” [8]. Breast cancer biomarkers can be classified regarding their usage as risk screening, prognostic, predictive and diagnostic and disease monitoring biomarkers [9,10]. Although current morphological features such as tumor size, histological type, cellular and nuclear characteristics, mitotic index, necrosis, vascular invasion, hormonal receptors and axillary tumor lymph node status are routinely used, they are not sufficient for reliable and early diagnosis of breast cancer. Fortunately, developments in molecular biology have led to the discovery of novel circulating biomarkers—DNAs, mRNAs, cell surface receptors, transcription factors, and secreted proteins—which have demonstrated to be extremely valuable tools for establishing reliable and early breast cancer diagnosis in a minimally invasive way [11].

In fact, nowadays almost all attention is focused on determining circulating breast cancer biomarkers in a wide variety of body fluids. They can derive from various molecular origins, including small DNAs (i.e., specific mutations in characteristic genes such as *BCRA1*), cell-free DNAs, messenger and microRNAs (mRNAs and miRNAs), proteins (soluble or membrane-associated proteins and glycoproteins), exosomes and tumor cells. Sensitive and rapid analytical methods for the reliable determination of these biomarkers in liquid biopsy samples are crucial since their level is useful for understanding the pathogenesis of disease prognosis, for early and reliable diagnosis and in the development of new therapeutic treatment regimens [12]. 

Although genomic (e.g., DNA sequencing, polymerase chain reaction (PCR) amplification detection, DNA microarray assay) and proteomic (conventional enzyme linked immunosorbent assays, ELISAs) methods are being extensively employed for the determination of circulating breast cancer biomarkers, they have limited multiplexing capabilities and involve multi-step, high-cost, and time-consuming processes demanding skilled people, which limits significantly their applicability for POC diagnosis [12]. Therefore, there is an urgent demand to develop portable, easy handling, time-efficient, cost-effective and quantitative tools for reliable determination of circulating biomarkers at different molecular levels. These analytical tools should be readily implemented in decentralized and resource-limited settings. These features can be fulfilled by using electrochemical biosensors. 

## 3. Electrochemical Biosensing of Circulating Breast Cancer Biomarkers

Electrochemical biosensing, based on the use of appropriate bioreceptors as recognition elements and electrochemical transduction to transform specific interactions into detectable electrical signals, has largely shown the ability of conventional technologies to address the deficiencies for the determination of circulating biomarkers related with breast cancer, providing unique advantages in terms of sensitivity and selectivity, low cost, simplicity of use and instrumentation, rapid responses and easy miniaturization and integration into portable and multiplexed platforms [10,12].

Most of the electrochemical biosensors reported so far for the determination of circulating breast cancer biomarkers are affinity biosensors, mainly immuno- and geno-sensors. They involve the immobilization of a specific recognition layer (antibody or nucleic acid)—able to recognize selectively a target biomarker of protein or genetic nature—onto the electrode surface and the interrogation of the electrochemical response before and after the affinity reaction with the target analyte happens. Bioreceptors used in the electrochemical biosensing of circulating biomarkers associated with breast cancer include specific single-stranded (ss) oligonucleotide strands of DNA/RNA nature (both linear and hairpin probes), aptamers, antibodies and much less frequently peptides. 

Apart from exploring high affinity bioreceptors, the advances in the use of screen-printed electrodes (SPEs), magnetic beads (MBs) and nanomaterials are playing major roles in the improvement of the analytical performance of electrochemical biosensing for the determination of circulating breast cancer biomarkers as well as in their miniaturization capability and in the reduction of the production costs [12]. 

The use of MBs improves significantly the electrochemical biosensing performance in terms of sensitivity, reproducibility, and reduced assay turnaround [13]. MBs provide a large surface area for both the immobilization of bioreceptors and bioreactions, offering efficient solid supports for the isolation, purification, and pre-concentration of target analytes in a straightforward way by means of an external magnetic field. Since MBs are dispersible in solution, upon modification with an appropriate receptor, they may greatly improve the capture efficiency of circulating breast cancer biomarkers [14]. Most importantly, the biosensing electrochemical methodologies involving MBs perform the biorecognition and transduction steps on different surfaces, thus minimizing the major problems associated with nonspecific adsorptions and therefore enhancing considerably the assay specificity and sensitivity.

SPEs—with low cost mass production—have been extensively used in the development of portable, miniaturized and low sample consumption electrochemical biosensors. Other interesting features of SPEs include the versatility for customizing their production and modification especially for specific and multiplexed applications. Moreover, their planar surface makes easy SPEs modification and their coupling with MBs through a localized magnetic field. All these unique features add up to produce their great potential in POC tests [12]. 

Since the performance of electrochemical biosensors depends strongly—apart from the morphology—on the amount, spacing and stability of the bioreceptors immobilized on the surface of the sensing device, the incorporation of a wide variety of unique nanomaterials into their fabrication with a large active surface area, abundant binding points, favorable catalytic activity, conductivity and biocompatibility, has been widely reported to improve sensitivity, selectivity, minimize interferences from complex biological matrices, and to prolong their stability, meeting the demands for detection of extremely low concentrated circulating biomarkers in scarcely treated samples [15,16]. These nanomaterials have been used mainly as electrode modifiers, advanced labels and redox mediators. 

Within this interesting context, this review overviews the main electrochemical biosensing strategies developed over the last five years for non-invasive breast cancer diagnosis. Only those approaches which demonstrated applicability in the analysis of liquid biopsies (mainly whole blood, serum and saliva) are discussed, and classified according the molecular level of the circulating breast cancer biomarker detected. Main involved bottlenecks and possible future research directions are also pointed out.

### 3.1. Electrochemical Biosensing of Gene Specific Mutations and miRNAs Associated with Breast Cancer in Biofluids 

Most genetic biomarkers used for non-invasive breast cancer diagnosis include the detection of specific mutations in certain genes and dysregulation measurement of specific miRNAs. Table 1 summarizes all the reported approaches involving electrochemical genosensors for circulating breast cancer genetic biomarkers. Most common gene mutations—all associated with the activation of breast cancer cells—imply BRCA1, BRCA2 and p53 genes [1]. 

BRCA1 is a human caretaker gene expressed in breast and other tissues’ cells. It encodes the BRCA1 protein involved in the prevention of fast and uncontrolled growth of cells. Mutations in this gene make the BRCA1 protein unable to repair damaged DNA leading in this way cells to grow uncontrollably and form tumors [1]. Indeed, mutations in the BRCA1 gene are responsible for approximately 40% of inherited breast cancer and specific mutations such as the BRCA1 5382insC mutation increase by 10 times the risk of getting breast cancer [1,2,17,18].

Regarding the detection of mutations in the BRCA1 gene, Benvidi et al. [19] described a simple, reproducible and impedimetric genosensor. The method involved the immobilization of a binary self-assembled monolayer composed of a thiolated specific capture DNA probe (BRCA1 5382 insC mutation detection) and 6-mercapto-1-hexanol (MCH) on a gold electrode. Using electrochemical impedance spectroscopy (EIS), the prepared biosensor provided a linear range within 1.0 × 10^−19^–1.0 × 10^−7^ M with a LOD of 4.6 × 10^−20^ M for the synthetic target and satisfactory results in the analysis of genomic DNA extracted from peripheral blood samples.

Another impedimetric DNA hybridization biosensor for the idenfication of BRCA1 mutations was developed by Wang et al. [2]. The biosensor implied the preparation of an antifouling glassy carbon electrode by highly cross-linking of polyethylene glycol films containing amine groups where gold nanoparticles (AuNPs) were self-assembled for further immobilization of a *BRCA1* related 19-mer DNA sequence. This approach allowed a linear range from 50.0 fM to 1.0 nM with a LOD of 1.72 fM for the synthetic target. The excellent sensitivity and antifouling properties shown by the hybrid PEGylated polymer–gold nanoparticle interface are responsible for the good analytical performance exhibited by the biosensor in the accurate determination across a broad clinically relevant concentration range in doped serum samples. 

miRNAs are nowadays considered to be non-invasive molecular biomarkers reliably linked to human cancers development and other relevant diseases. Recently, accumulative evidence indicates that miRNAs are highly correlated with cancer initiation, oncogenesis, and tumor response to treatments [20]. Moreover, they are perfectly stable in serum/plasma and the levels of some circulating miRNAs (typically in the range of 200 aM to 20 pM [6]) have been found to be specifically dysregulated in association with breast cancer so that they can be used as suitable biomarkers for its early detection [7]. Breast cancer miRNAs exhibit oncogene or tumor suppressor roles, depending on their target genes. Amplification of chromosomal regions encoding oncogenic miRNAs (such as miR-21 and miR-155), which inhibit tumor suppressor genes, results in the up-regulation of miRNAs and in the subsequent silencing of these genes, which is highly associated with cancer development. On the other hand, tumor-suppressor miRNAs (such as miR-205) are usually located in chromosomal fragile regions and different alterations such as deletions or mutations of these regions leads to a reduction or loss of expression of these miRNAs and subsequent up-regulation of their target oncogenes. 

**Figure 1:**
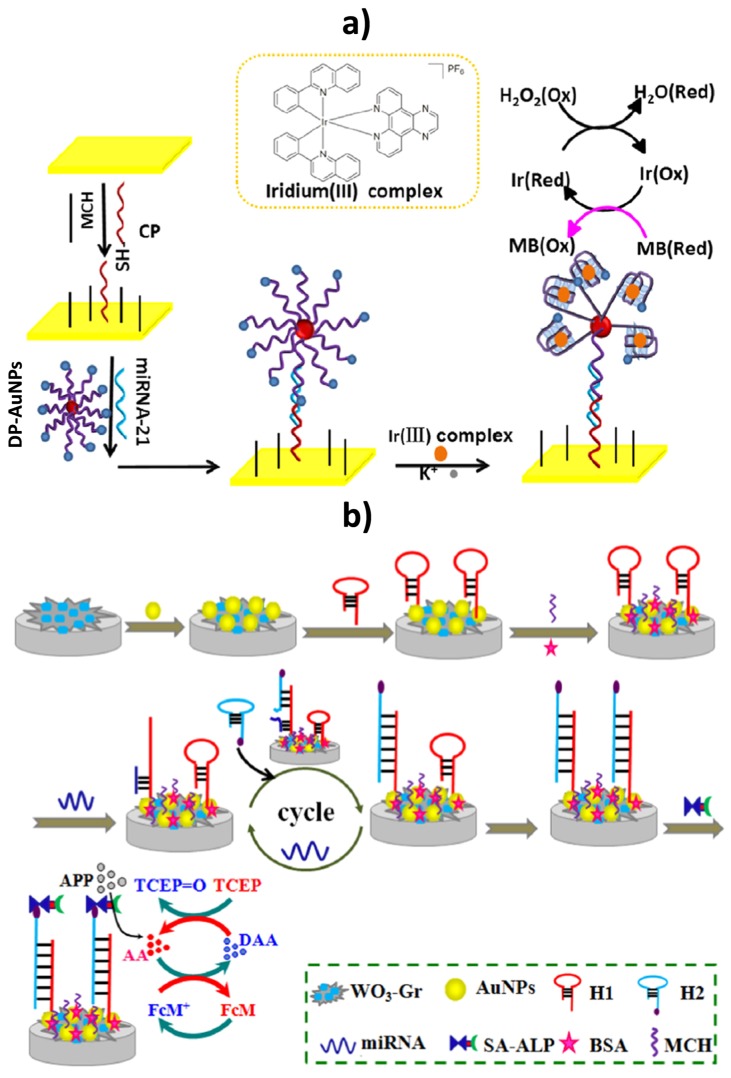
Schematic displays of the (**a**) electrochemical biosensing strategies developed for miR-21 determination based on the use of an iridium(III) complex which selectively and stably interacted with G-quadruplex DNA acting as a peroxidase-like mimic; (**b**) catalyzed hairpin assembly target coupled with recycling and enzyme signal amplification. Reprinted from [21] (**a**) and [20] (**b**) with permission.

Currently, electrochemical nucleic acid-based biosensing strategies provide attractive alternatives able to overcome the limitations of conventional strategies for miRNAs determination (microarrays, Northern Blotting, Reverse Transcriptase PCR (RT-PCR) and In Situ Hybridization (ISH)). At present, the increasing demand for the ultra-low detection of miRNAs with electrochemical techniques is driving the enhancement of sensitivity by selecting different signal amplification strategies involving enzymes, nanomaterials and other amplification systems. The basis and main characteristics of the electrochemical biosensing approaches reported so far for the determination of breast cancer-related circulating miRNAs in biofluids are summarized in Table 1. Illustrating examples of these attractive and sensitive approaches are schematically displayed in Figure 1 for the determination of miR-21 [20,21]. Miao et al. developed a sensitive redox and catalytic “all-in-one” sensing platform for miRNA-21 detection by employing the G-quadruplex DNA species and an iridium(III) complex as a peroxidase-like mimic to catalyze the reduction of H_2_O_2_ and methylene blue (MB) as redox mediator (Figure 1a) [21]. In this approach, the target miRNA-21 was sandwiched between a capture probe (CP) immobilized onto the gold electrode surface and a methylene blue (MB) labeled G-rich detection probe (DP) modified onto AuNPs. Upon the addition of K^+^, the structure of DP changed to a G-quadruplex and the iridium(III) complex could selectively interact with the G-quadruplex, catalyzing the reduction of H_2_O_2_, in the presence of MB as electron mediator. Under optimal conditions, the increase in the MB reduction peak measured by CV was proportional to miRNA concentration in the range from 5.0 fM to 1.0 pM, with a LOD of 1.6 fM. This biosensing platform provided satisfactory results for miRNA-21 determination in spiked human serum samples.

An ultrasensitive electrochemical biosensor for miRNA-21, based on tungsten oxide-graphene composites (WO_3_-Gr) coupling with catalyzed hairpin assembly target recycling and enzyme signal amplification, was developed by Shuai et al. [20]. A thiolated hairpin capture probe (H1) was immobilized on a GCE modified with WO_3_-Gr composites and AuNPs (Figure 1b). In the presence of target miRNA, H1 opened its hairpin structure and target miRNA was displaced by another stable biotinylated hairpin DNA (H2) and released back to the sample solution for next cycle. After the cyclic process a lot of conjugates of streptavidin-alkaline phosphatase (SA-ALP) were immobilized on the electrode by binding to the large amount of biotinylated H1-H2 duplexes. For the DPV detection an induced electrochemical-chemical-chemical (ECC) redox cycling triggered by the enzymatic product ascorbic acid (AA) using ferrocene methanol (FcM) and tris(2-carboxyethyl)phosphine (TCEP) as the redox mediator and the reducing reagent, respectively, was used. This approach detected target miRNA-21 down to 0.05 fM with a linear range from 0.1 fM to 100 pM, discriminated target from mismatched miRNAs with a high selectivity and provided results in agreement with RT-PCR in the analysis of human serum samples of breast cancer patients.

### 3.2. Electrochemical Biosensing of Breast Cancer Protein Circulating Biomarkers

Electrochemical biosensors have been widely explored for the determination of breast cancer circulating biomarkers of protein nature (soluble or membrane-associated proteins and glycoproteins) mainly using antibodies and aptamers or a combination of both as biorecognition elements. Therefore, the selected examples in this section will be sub-classified according to the type of bioreceptors used.

#### 3.2.1. Electrochemical Aptasensors for Breast Cancer Protein Circulating Biomarkers

A folding-based electrochemical DNA aptasensor was reported by Zhao et al., for the detection of vascular endothelial growth factor (VEGF) directly in blood serum and whole blood [29]. The method involved the immobilization of an aptamer, dually labeled with thiol and methylene groups, on a gold electrode. The electrochemical signal of MB, measured by alternating current voltammetry (ACV), was enhanced in the presence of VEFG due to the formation of a rigid stem-loop structure in the aptamer which brought the MB label closer to the electrode. This reusable aptasensor allowed a linear range ranging from 50 pM to 0.15 nM and readily detected 50 pM VEGF directly in 50% blood serum. This simple “signal-on” sensor architecture did not need competitive binding and target-induced displacements and circumvented the general disadvantages of “signal-off” sensors, in which only 100% signal suppression can be realized even under optimized conditions.

**Figure 2:**
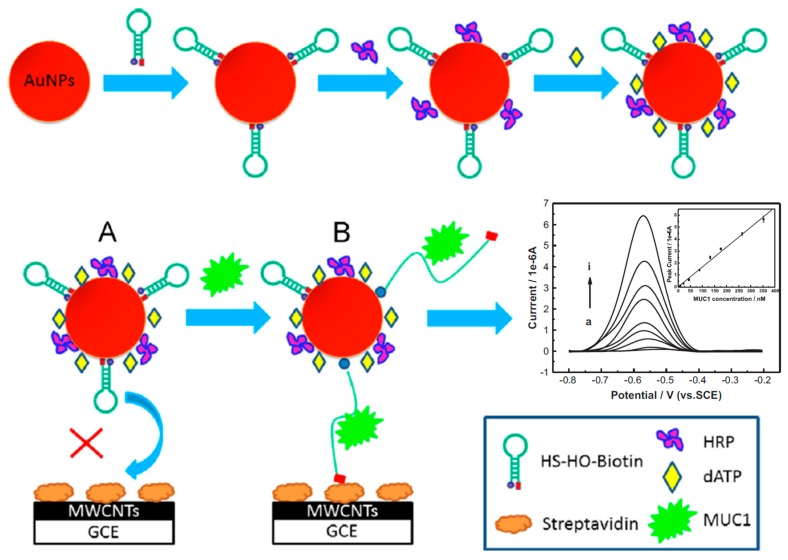
Schematic illustration of the electrochemical method developed for MUC1 determination using HO-AuNP-HRP conjugates at a Strept-MWCNTs-GCE. Real DPV responses obtained for MUC1 standards of different concentrations. Reprinted from [30] with permission.

Hu et al. [30] developed a biosensing platform for mucin 1 protein (MUC1) determination using a hairpin oligonucleotide (HO) aptamer dually labeled with thiol and biotin, AuNPs, and enzyme signal amplification. Hairpin aptamers and horseradish peroxidase (HRP) were immobilized on the AuNPs through thiol chemistry and adsorption, respectively, to yield the HO-AuNP-HRP conjugates used as labels. After biorecognition between the HO and MUC1, the hairpin aptamer, initially in a “closed” state, was disrupted and the thus exposed biotin moiety was used to capture the conjugates on an electrode modified with MWCNTs and streptavidin (Strept) (Figure 2). The reduction of 2,3-diaminophenazine (DAP)—the reaction product generated by the enzymatic oxidation of o-phenylenediamine in the presence of H_2_O_2_, was monitored by differential pulse voltammetry. The approach provided a good linear correlation range (8.8–353.3 nM), a LOD of 2.2 nM and promising results in the analysis of spiked serum samples.

**Figure 3:**
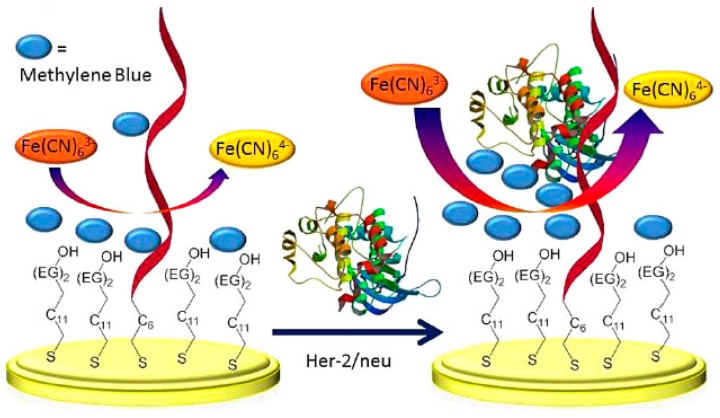
Schematic display of the aptasensor for HER2 determination. Reprinted from [31] with permission.

Very recently, a label-free aptameric sensor for human epidermal growth factor receptor 2 (HER2) has been reported by Salimiam et al. [31]. This aptasensor was based on the co-adsorption of a specific thiolated aptamer sequence on gold together with a C11 alkanethiol bearing two ethylene glycol (EG)_2_ head groups which prevented non-specific adsorptions of non-target proteins (Figure 3). The measurement of the electrocatalyzed reduction of ferricyanide redox indicator by MB electrostatically interacting with negatively charged HER2 allowed the detection of 10^−12^–10^−8^ M HER2 in 1% (*v*/*v*) serum.

#### 3.2.2. Electrochemical Immunosensors for Protein Circulating Biomarkers

The construction of a reagentless and mediatorless immunosensor for the determination of CA15-3 was proposed by Li et al. [32]. The immunosensing platform utilized a GCE modified with a composite material containing CNTs and core-shell organosilica@chitosan nanospheres together with two successive layers of Pt nanoclusters, the first one with glucose oxidase (GOD) and the second with the capture antibodies adsorbed. The immobilized GOD showed direct electron transfer. The peak current corresponding to the cofactor oxidation measured by cyclic voltammetry decreased linearly with the increasing logarithm of CA15-3 concentration from 0.1 to 160 U mL^−1^ with a LOD of 0.04 U mL^−1^. Moreover, results provided in the analysis of human serum samples agreed with those obtained using a conventional ELISA methodology. 

Munge et al. [33] described an ultrasensitive sandwich immunosensor for the determination of interleukin 8 (IL-8). The immunosensor construction involved the use of a glutathione-protected gold nanoparticle sensor surface modified with the capture antibody and 1.0 μm superparamagnetic beads massively loaded with the detector antibody and 500,000 HRP labels attached through avidin–biotin interaction. A LOD of 1 fg mL^−1^ (100 aM) in human serum was achieved.

An electrochemical immunosensor for carcinoembryonic antigen (CEA) using a magnetic nanocomposite with redox activity and good biocompatibility and gold-graphene nanolabels was developed by Chen et al. [34]. The redox-active magnetic nanostructures were synthetized by combination of a magnetic nanocore, a layer of electroactive poly(o-phenylenediamine) (PPD), and a silver metallic shell. The as-prepared nanocomposite offered good adsorption properties for the attachment of an anti-CEA antibody and good redox behavior to facilitate and modulate the way it was integrated into a magnetic carbon paste electrode. The doped PPD acted not only as a cross-linkage but also as a mediator for the electron transfer. The target antigen was sandwiched between the immobilized anti-CEA antibody and nanogold-patterned graphene oxide (AuNP-GO), conjugated with HRP-labeled anti-CEA, and was used as trace tags. By measuring by DPV the cathodic current corresponding to the enzymatic reoxidation of PPD in the presence of H_2_O_2_, the method allowed detection of CEA at a 1.0 pg mL^−1^ concentration level and successful applicability in real serum specimens. The reported strategy has the following main merits: (i) an efficient encapsulation of the redox electroactive species into the magnetic nanocomposites that facilitated the electrochemical measurement and avoided their contamination in the detection solution; (ii) the trapezoidal and semi-ladder structures synthesized with the PPD polymers provided a large surface coverage for the patterning of silver nanoparticles; (iii) two-dimensional graphene nanosheets were used for the immobilization of AuNPs and multiple biomolecules, thus enhancing the possibility of antigen–antibody interaction.

Elshafey et al. [35] developed a sensitive label-free impedimetric immunosensor for the detection of the epidermal growth factor receptor (EGFR) using protein G-modified AuNPs electrodeposited on a gold electrode modified with a cysteamine self-assembled monolayer (SAM). Protein G was attached to the amine-terminated SAM modified electrodes using a 1,4-phenylenediisothiocyanate linker. The capture antibodies were immobilized on the modified AuNPs through their non-antigenic Fc regions. The electrodeposition of AuNPs increased the electrochemical active area by a factor of approximately 3 while protein G enabled well oriented EGFR antibodies immobilization. The impedimetric transduction allowed a wide dynamic range (1 pg mL^−1^–1 μg mL^−1^) and LODs of 0.34 and 0.88 pg mL^−1^ in phosphate-buffered saline (PBS) and human plasma, respectively, to be obtained. In addition, excellent recoveries in spiked human plasma and mouse brain tissue homogenate were achieved. 

Another impedimetric biosensor was reported for the determination of human epidermal growth factor receptor 3 (HER3) by covalent immobilization of the capture antibody on a gold electrode modified with a 4-aminothiophenol SAM using glutaraldehyde as cross-linking agent [36]. The immunosensor exhibited a linear detection range of 0.4–2.4 pg mL^−1^ and feasibility to the analysis of artificial serum samples.

Marques et al. [37] proposed an electrochemical immunosensor for the analysis of the HER2 extracellular domain (ECD) in human serum based on a sandwich assay implemented at SPEs modified with AuNPs. The biotinylated detector antibody was labeled with a Strept-ALP conjugate and the metallic silver generated in the presence of the enzymatic substrate (3-indoxyl phosphate, 3-IP) and silver ions was detected by linear sweep anodic stripping voltammetry. Under the optimized conditions, a calibration curve (*i_p_* vs. log[HER2 ECD]) between 15 and 100 ng mL^−1^ and a LOD of 4.4 ng mL^−1^ (below the established cut-off value of 15 ng mL^−1^) were achieved. 

**Figure 4:**
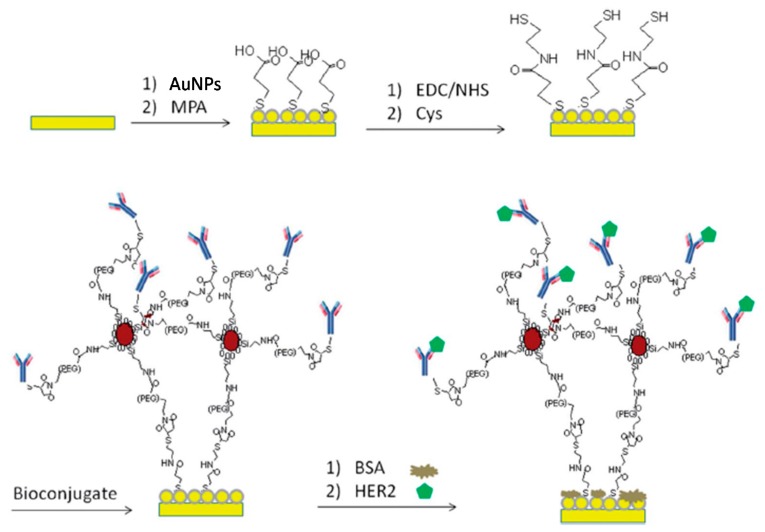
Graphical illustration of the label-free immunosensor developed for HER2 determination involving the use of antiHER2–Fe_3_O_4_ NPs bioconjugates immobilized onto a Cys/MPA/AuNPs/AuE platform. Reproduced from [38] with permission.

A label-free immunosensor for the determination of HER2 was reported by Emami et al. In this strategy, thiolated capture antibodies were attached to PEG–maleimide-coated iron oxide nanoparticles (Fe_3_O_4_ NPs) to form stable highly loaded bioconjugates which were subsequently laid over a modified gold electrode surface [38]. AuNPs were electrodeposited on the gold electrode and further modified with a 3-mercaptopropionic acid (MPA) SAM whose carboxylic groups were then activated with 1-ethyl-3-(3-dimethylaminopropyl)-carbodiimide/N-hydroxysuccinimide (EDC/NHS) to attach cysteamine (Cys). As shown in Figure 4, thiol groups in Cys were then used to attach the antiHER2–Fe_3_O_4_ NPs bioconjugates. While the nanoparticles facilitated electron transfer with redox probes, the bioconjugates also increased the sensitivity of the method by allowing the immobilization of a high loading of antibodies on the electrode surface while keeping them long enough to allow their efficient interaction with the target biomarker. By measuring the decrease in the DPV response in the presence of Fe(CN)_6_^3−/4−^, the immunosensor showed two different linear ranges (0.01–10 ng mL^−1^ and 10–100 ng mL^−1^) and provided a LOD of 0.995 pg mL^−1^. In addition, the reliability of this impedimetric immunosensing method was proved by successful quantization of HER2 levels in patients’ serum samples. 

**Figure 5:**
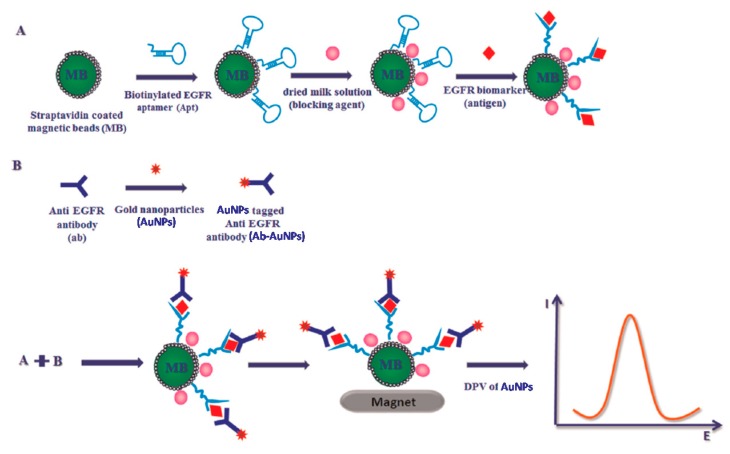
Schematic display of the electrochemical Apt/Ab sandwich immunosensor developed for the detection of EGFR using Apt-modified MBs and Ab-modified AuNPs. Reprinted from [14] with permission.

Ilkhani et al. [14] reported for the first time the development of an electrochemical aptamer/antibody (Apt/Ab) sandwich immunosensor for the detection of EGFR. Strept-coated MBs modified with a biotinylated anti-human EGFR aptamer were employed as capture microcarriers whereas AuNPs conjugated with detector antibodies were used as signaling probes (Figure 5). The extent of the complexation was evaluated by DPV after dissolution of the AuNPs in acidic medium. A linear range from 1 to 40 ng mL^−1^ EGFR and a LOD of 50 pg mL^−1^ were reported. This method combined a sandwich assay configuration for high specificity, MBs for fast separation, and electrochemical method for cost-effective and sensitive detection, and showed usefulness for the successful discrimination between healthy and breast cancer patient serum samples by means of the EGFR content.

A label-free electrochemical CEA immunosensor using the measurement of silver oxidation on a cryogel electrode was described by Samanman et al. [39]. This method combined the advantages of graphene (unique electrical conductivity and enlarged active surface area), AuNPs (excellent biocompatibility and large surface area) and chitosan (good adhesion, non-toxicity and abundant reactive amino groups) with the ease of preparation of a cryogel (better electron transfer and larger surface area) coupled to silver deposition on an Au electrode. Indeed, the use of this cryogel-electrode demonstrated a 1.7 times larger sensitivity and 25 times lower LOD than the non-modified electrode. The decrease of the silver oxidation peak current measured by CV was proportional to the CEA concentration in the range from 1.0 × 10^−6^ to 1.0 ng mL^−1^ and allowed a LOD of 2.0 × 10^−7^ ng mL^−1^. Moreover, the results provided in the analysis of human serum samples were in agreement with those obtained by the ELISA conventional methodology. 

Xu et al. [40] reported another electrochemical immunosensor for CEA using poly(o-phenylenediamine) nanospheres (PPDNSs) as support for the immobilization of a specific HRP-labeled antibody (HRP-anti-CEA) on a pretreated GCE using glutaraldehyde as a crosslinker agent. In the presence of the target CEA, the electrochemical reduction of H_2_O_2_ was inhibited and the catalytic current measured by DPV in the absence of an external electron mediator was proportional to the CEA concentration in the 0.01 to 60 ng mL^−1^ range, with a LOD of 3.2 pg mL^−1^. The method was applied to the analysis of human serum samples with results in agreement with those obtained using a commercial ELISA kit. The most interesting feature of this approach is the 20 min assay time. 

**Figure 6:**
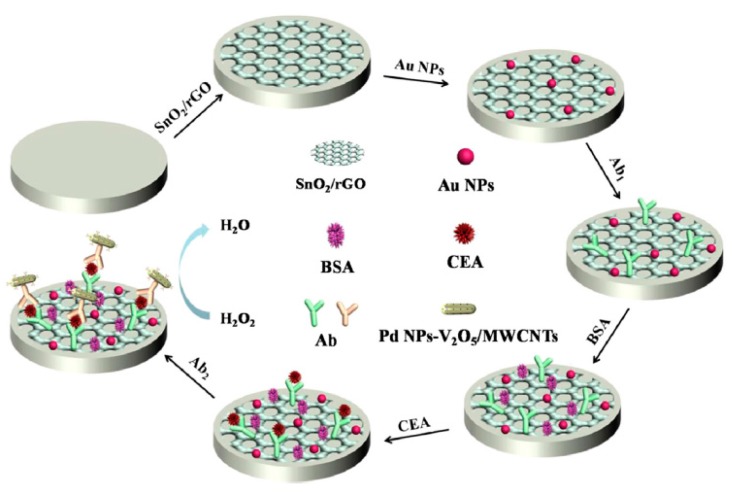
Schematic display of the developed non-enzymatic sandwich immunosensor for the determination of CEA using SnO_2_/rGO/AuNPs and PdNPs–V_2_O_5_/MWCNTs nanocomposites as scaffold and advanced labels, respectively. Reprinted from [41] with permission.

A sandwich-type non-enzymatic electrochemical immunosensor for CEA was developed by immobilizing the capture antibody onto a GCE modified with a nanocomposite of stannic oxide/reduced graphene oxide (SnO_2_/rGO) and AuNPs [41]. The captured CEA was labeled with PdNPs-vanadium pentoxide (V_2_O_5_)/MWCNTs and conjugated with secondary antibodies (Ab_2_) (Figure 6). While the nanocomposite provided a large specific surface area for antibodies immobilization and accelerated the electron transfer, the PdNPs–V_2_O_5_/MWCNTs demonstrated an excellent catalytic activity towards H_2_O_2_ reduction. This nanostructured immunosensor showed a wide linear range (from 0.5 pg mL^−1^ to 25 ng mL^−1^), a LOD of 0.17 pg mL^−1^ and good recoveries in the analysis of human serum samples.

Eletxigerra et al., developed sandwich amperometric magnetoimmunosensors for the determination of HER2 [42] and estrogen receptor α (ERα) [43] in human serum. In both cases, capture antibodies were covalently immobilized on carboxylic acid modified MBs and the captured target protein sandwiched with HRP-labeled (HER2) or biotin-labeled (ERα) detector antibodies, in this later case further conjugated with a Strept-HRP polymer. The sandwich magneto-immunoconjugates were magnetically captured on the surface of screen-printed carbon electrodes, and the cathodic current measured by amperometry in the presence of H_2_O_2_ and hydroquinone was proportional to the concentration of the target proteins. Both magnetoimmunosensors provided linear ranges (0.1–32.0 ng mL^−1^ and 63–2000 pg mL^−1^ for HER2 and ERα, respectively) and LODs (26 pg mL^−1^ and 19 pg mL^−1^ for HER2 and ERα, respectively) within the clinical relevant ranges and demonstrated successful applicability in the analysis of spiked (ERα) and non-spiked (HER2) human serum samples. Interestingly, both magnetoimmunosensors also demonstrated capability to discriminate intact breast cancer cells with respect to the expression level of the target receptor and, therefore, feasibility to perform the determination of circulating tumor cells. 

**Figure 7:**
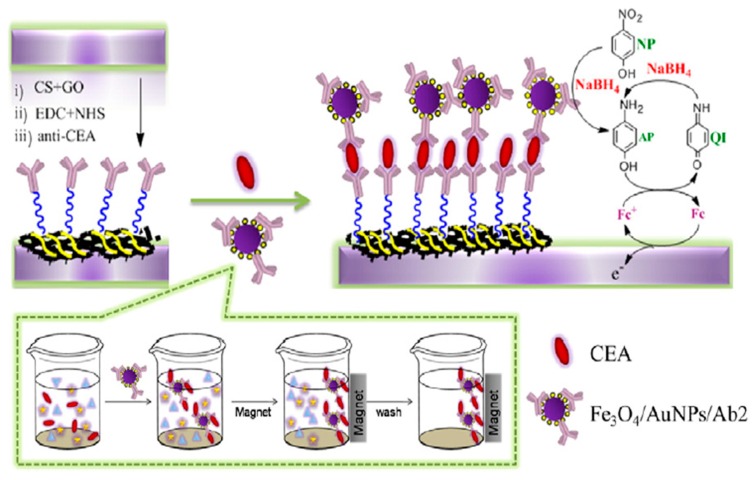
Schematic illustration of the preparation of an immunosensor for CEA employing GO/CS–Fc as scaffold for the immobilization of capture antibodies and Fe_3_O_4_/AuNPs functionalized detector antibodies. The electrochemical reactions involved in the affinity reaction monitoring are also shown. Reprinted from [44].

An attractive sandwich electrochemical immunosensor for the determination of CEA has been described using an enzyme-free signal amplification strategy employing graphene oxide/chitosan–ferrocene (GO/CS–Fc) as a matrix for capture antibodies’ immobilization and Fe_3_O_4_/AuNPs functionalized detector antibodies [44]. The sensitive determination of CEA was achieved by catalytic reduction of p-nitrophenol to electroactive p-aminophenol using the gold-nanocatalyst labels. Then, p-aminophenol is oxidized to p-quinone imine by electrochemically generated ferrocenium ion. Finally, NaBH_4_ reduces quinone imine to aminophenol, which can be re-oxidized and monitored by CV (Figure 7). The use of Fe_3_O_4_/AuNPs labeling provoked a 10-fold increase in the detected signal compared to the immunosensor prepared without labels, a determination range of 0.001–30 ng mL^−1^, and a LOD of 0.39 pg mL^−1^. The usefulness of the immunosensor was evaluated in spiked human serum samples.

A label-free immunosensor for the rapid detection of CEA was reported using Pd–Ir bimetallic nanoparticles as catalyst [45]. A specific antibody was absorbed on a GCE modified with the Pd–Ir bimetallic nanoparticles using chitosan. The current response, measured by amperometry, was attributed to the catalytic activity of Pd–Ir nanoparticles for the reduction of H_2_O_2_, and showed a linear decrease with the concentration of CEA over the 0.05 to 50 ng mL^−1^ range, allowing a LOD of 0.017 ng mL^−1^. Moreover, results in the analysis of spiked human serum samples demonstrated the practical applicability of this sensor. 

**Figure 8:**
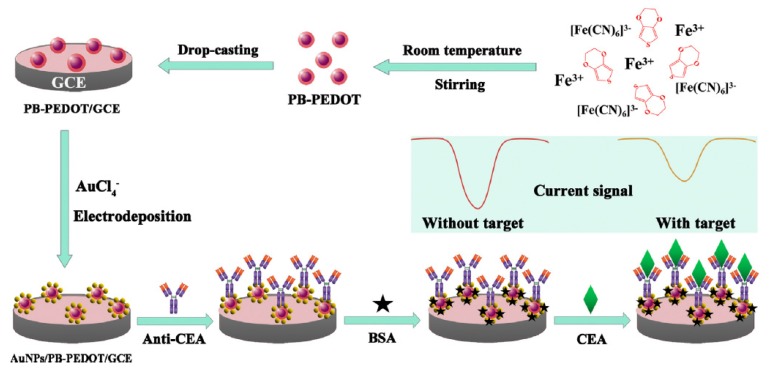
Immunosensor fabricated for the determination of CEA using AuNPs/PB-PEDOT nanocomposite both as electron mediator and 3D matrix for the immunosensor fabrication. Reprinted from [46] with permission.

Yang et al. [46] have recently reported a novel label-free electrochemical immunosensor for CEA involving a AuNPs-decorated Prussian blue-poly(3,4-ethylenedioxythiophene) (AuNPs/PB-PEDOT) nanocomposite with a 3D hierarchically porous structure which exhibited favorable microenvironment and biocompatibility together with an excellent redox activity (Figure 8). PEDOT was used to facilitate interfacial electron transfer and to improve the stability of PB while the AuNPs were employed to immobilize the capture antibody. The strong redox activity of the 3D AuNPs/PB-PEDOT nanocomposite—hindered after CEA recognition due to the insulating effect of the antigen-antibody complex—was measured by DPV. This approach, in which AuNPs/PB-PEDOT was used both as electron mediator and 3D matrix for immunosensor fabrication, offered linearity with the concentration of CEA ranging from 0.05 to 40 ng mL^−1^ and a LOD of 0.01 ng mL^−1^. The usefulness of the immunosensor for clinical applications was demonstrated by analyzing the CEA endogenous content in human serum samples demonstrating good correlation with the ELISA method.

#### 3.2.3. Electrochemical Peptide Biosensor for Breast Cancer Protein Circulating Biomarkers

Kemal [11] developed an impedimetric biosensor for the determination of VEGF using vascular endothelial growth factor receptor-1 (VEGF-R1) as the recognition element, covalently immobilized onto a gold electrode modified with a MPA SAM. This immunosensing method provided a linear response from 10 to 70 pg mL^−1^ and demonstrated the feasibility for the analysis of spiked artificial human serum samples. 

It is worth it to mention here that the detection of some membrane-associated glycoproteins such as HER2 and MUC1 has been used also to develop cytosensors for breast cancer circulating cells, as is discussed in the next section.

### 3.3. Electrochemical Biosensing of Circulating Breast Cancer Cells

The simple, rapid, sensitive, and specific detection of cancer cells plays a pivotal role in the diagnosis and prognosis of breast cancer [47]. Circulating tumor cells (CTCs) are cells shed by primary tumors that travel through the bloodstream and serve as catalysts for the growth of metastatic tumors. Therefore, the ability to capture and count these cells is an extremely important goal in order to eliminate the need for more invasive sampling methods also leading to earlier disease control. However, this is a very challenging task given the low abundance of CTCs (~10 cells mL^−1^) compared with erythrocytes and leukocytes (~10^9^ and 10^6^ per mL, respectively) in blood [4].

Zhu et al. [5] developed an electrochemical biosensor for the determination of both HER2 protein and HER2-overexpressing breast cancer cells. The sensor was constructed by the covalent immobilization of a specific capture antibody onto a nanocomposite layer composed of self-assembled 2,5-bis(2-thienyl)-1H-pyrrole-1-(p-benzoic acid) (DPB) on AuNPs. Hydrazine−AuNPs−aptamer bioconjugates were used as labels to reduce and selectively deposit silver ion for signal amplification. The electrochemical oxidation of deposited silver was monitored by square wave stripping voltammetry. The immunosensor showed a dynamic range from 0.1 pg mL^−1^ to 10 ng mL^−1^ and a LOD of (0.037 ± 0.002) pg mL^−1^ of HER2 protein in 25-fold diluted human serum and was able to differentiate between HER2-positive (SK-BR-3) and HER2-negative breast cancer cells (MCF-7 and Hela) and to detect 26 SK-BR-3 cells mL^−1^ in human serum samples. The AuNP-promoted silver enhancement and the combination of a monoclonal antibody and a specific aptamer were considered responsible for the high sensitivity and selectivity, respectively, of this biosensing approach.

An electrochemical cytosensor for highly selective and sensitive detection of cancer cells by using folate-linked DNA probes was reported by Zhao et al. [48]. The methodology was based on the digestion of folate-linked DNA probes immobilized on an electrode surface by exonuclease I. In the presence of folate receptor-positive cells (human breast cancer MCF-7 cells), the probes were protected from digestion upon the binding with folate receptor that was over-expressed on the cell surface. The chronocoulometric response of [Ru(NH_3_)_6_]^3+^ bonded electrostatically to the probe immobilized on a gold surface and was used to determine the target cells. The charge density increased with the concentration of the target cells because the DNA probe bound to cells was protected from ExoI-catalyzed digestion and higher amounts of [Ru(NH_3_)_6_]^3+^ were bound to the intact DNA probe. The proposed method showed a linear detection of MCF-7 cells in a wide range from 10^2^ to 10^6^ cells mL^−1^ with a low LOD of 67 cells mL^−1^. 

Moscovici et al. [4] developed a microfabricated glass chip electrochemical device able to specifically count 125 prostate cancer cells (DU145 cells) in 15 min in either a complex media with serum or a mixed cell population containing non-target cells. The device featured a gold electrode array with tunable sensor surface areas, which were modified with a capture antibody specific for Epithelial cell adhesion molecule (EpCAM, anti-EpCAM antibody) to selectively count prostate cancer cells using the differential pulse voltammetric response in the presence of [Fe(CN)_6_]^3−/4−^. The microfabricated glass chip device provided rapid, label-free electrochemical detection of prostate cancer cells with high sensitivity and selectivity. 

**Figure 9:**
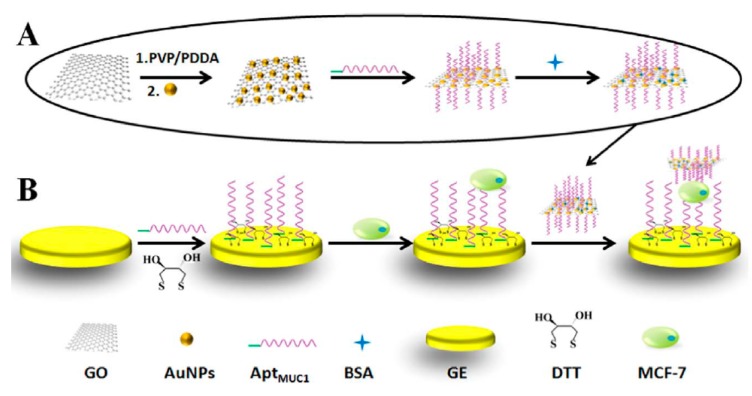
Schematic illustration of the sandwich aptasensors developed for MCF-7 determination using (**A**) a polydA-aptamer functionalized AuNPs/GO hybrid and (**B**) a polyadenine (polydA)-aptamer modified gold electrode. Reprinted from [47] with permission.

A sandwich electrochemical aptasensor was developed for the label-free and selective detection of breast cancer cells (MCF-7) using a polyadenine (polydA)-aptamer modified gold electrode and a polydA-aptamer functionalized AuNPs/GO hybrid (Figure 9) [47]. The decrease in the [Fe(CN)_6_]^3−/4−^ DPV peak current in the presence of the sandwich system was employed to quantify the breast cancer cells. The aptamer used in this work was against the MUC1 protein, which is overexpressed in MCF-7 cells. The polydA modified aptamer was immobilized on both bare gold electrode and GO modified with AuNPs due to the intrinsic affinity between multiple consecutive adenines of polydA sequences and this metal. The aptasensor demonstrated a high selectivity in discriminating MCF-7 cells against non-tumorigenic cells and other cancer cells, and provided a linear range of 10–10^5^ cells mL^−1^, a LOD of 8 MCF-7 cells mL^−1^, and successful applicability to the detection of 10-times diluted human serum samples spiked with the target cells.

### 3.4. Electrochemical Biosensing for Multiple Determination of Circulating Breast Cancer Biomarkers

Due to population variations in the expression of a single biomarker, the lack of totally specific biomarkers, and the multiple complex molecular events involved in breast cancer development and prognosis, the detection of a single biomarker does not support reliable diagnosis and prognosis of this neoplastic disease [49]. Accordingly, multiplexed detection of carefully selected biomarker candidates should lead to an earlier, more reliable and personalized breast cancer diagnosis, free of false positives [50,51]. Therefore, the development of electrochemical platforms able to detect biomarkers at the genetic (DNAs), regulatory (RNAs), functional (proteins) and metabolic (small molecules) levels is highly desirable [49]. It is worth it to remark at this point that, in comparison with conventional methods used for the determination of cancer biomarkers, electrochemical biosensors offer a great versatility to be translated for detection at different molecular levels and adapted to multiplexed strategies [52,53]. Some attractive electrochemical platforms have been reported until now for the multiplexed determination of circulating breast cancer biomarkers of the same or different molecular level.

Wei et al. [54] developed integrated electrochemical sensors for the determination of IL-8 mRNA and IL-8 protein in the same assay by immobilizing the biotinylated specific bioreceptors—hairpin probe sequence and antibody, respectively—onto a 16 gold electrodes-array chip modified with a conducting polymer incorporating Strept-dendrimer nanoparticles to improve the biocompatibility. Chronoampreometric measurements using the H_2_O_2_/3, 3, 5, 5-tetramethylbenzidine system allowed obtaining LODs of 3.9 fM and 7.4 pg mL^−1^ for IL-8 mRNA and IL-8 protein, respectively, in saliva samples. The biosensor was applied to the determination of both target biomarkers in 28 cancer and 28 matched control saliva samples.

An MBs-based dual amperometric disposable platform for the direct determination of IL-8 protein and IL-8 mRNA was also developed by Torrente-Rodríguez et al. [55] involving the use of functionalized MBs (Strept-MBs and HOOC-MBs), specific antibodies against IL-8 protein, a specific biotinylated hairpin DNA sequence for IL-8 mRNA and amperometric detection at disposable screen-printed dual carbon electrodes (SPdCEs). This method exhibited high selectivity and sensitivity for the target analytes yielding detection limits of 0.21 nM (IL-8 mRNA) and 72.4 pg mL^−1^ (IL-8 protein) in undiluted saliva samples. The dual amperometric biosensor was successfully applied to the direct determination of these two biomarkers in raw saliva samples spiked with known concentrations of synthetic IL-8 mRNA. 

**Figure 10:**
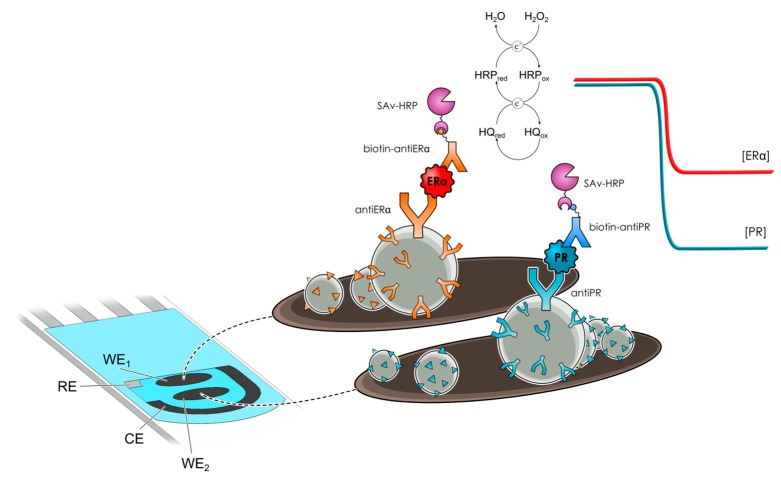
Schematic display of a disposable dual magnetoimmunosensor for the simultaneous detection of PR and ERα. Reprinted from [56] with permission.

Eletxigerra et al. [56] developed a dual scaffold—by coupling two previously developed single strategies—for the simultaneous determination of progesterone receptor (PR) and ERα in cellular lysates and spiked human serum samples at SPdCEs. The biosensor design implied the use of two different batches of functionalized MBs bearing HRP-labeled sandwich immunocomplexes specific for each protein receptor which were magnetically captured on the corresponding working electrode of the SPdCE (Figure 10). Amperometric detection of the catalytic current produced upon H_2_O_2_ addition using HQ in solution as redox mediator was employed to monitor each receptor concentration providing LODs at the level of low pg mL^−1^ for both receptors. The usefulness of the dual immunosensing platform was evaluated by analyzing spiked human serum and cancer cell lysates. Results obtained demonstrated the reliability of the approach to accurately determine the endogenous content of such biomarkers in these minimally pretreated complex samples without any matrix effect and the absence of significant cross-talking between the adjacent working electrodes. 

## 4. General Considerations, Challenges and Future Prospects

The previous sections in this review article provide useful insights into the latest advances and current tendencies in the electrochemical biosensing strategies reported so far for circulating breast cancer biomarkers with applicability in liquid biopsies analysis. The highlighted contributions describe the development and preliminary applicability of versatile electrochemical bioscaffolds for determining circulating breast cancer biomarkers at different molecular levels: gene mutations, miRNAs, mRNAs, proteins and even intact cells using single or multiplexed methods although still mainly in spiked serum and saliva samples. The wide variability of the selected approaches demonstrates the possibility of using different bioreceptors, bioassay formats and amplification strategies in connection with different electrode substrates and electrochemical techniques. It is worth mentioning that the variable complexity of the reported methodologies, ranging from a wide number of quite simple label-free approaches, such as signaling mechanism linked to target-induced conformational change of specific aptameric probes, to more complicated but extremely attractive strategies. Although most amplification methods make use of nanomaterials and/or enzymes, other less explored strategies based on the use of DNA concatamers and ECC redox cycling, nuclease assisted target recycling and target catalyzed hairpin assembly have also been proposed. 

Considering the high sensitivity required for the determination of circulating breast cancer biomarkers, and the remarkable current progress made in bioconjugation and nanotechnology techniques, most of the reported biosensing strategies have been developed in connection with the use of different nanomaterials taking advantage of the unique properties they offer when employed as electrode modifiers (AuNPs, CNTs, Pt NCs, SnO_2_/rGO, PPDNSs, organosilica@chitosan nanospheres, graphene, WO_3_), as signaling carriers (AuNPs, PdNPs–V_2_O_5_/MWCNTs, Fe_3_O_4_ NPs), or even as artificial enzymes (Pt NCs and Pd–Ir and PB NPs). Most of the reported methods have been developed using integrated formats, both at conventional and disposable electrodes. 

Taking into account the number of strategies described so far in this field, the determination of circulating breast cancer protein and miRs biomarkers have shown to be of special relevance. Other less explored but extremely interesting biosensing methods have been described recently for the determination of intact circulating breast cancer cells. All the compiled approaches have demonstrated compatibility with the clinical relevance range of the target biomarker and to be suitable for practical purposes through the analysis of real samples.

When electrochemical techniques are compared with optical techniques, which are also commonly used in biosensing of circulating breast cancer biomarkers, it can be said that the former have been more and more used in recent years because they are able to overcome some limitations of optical techniques in terms of portability, cost and compatibility with POC devices. These features make electrochemical biosensors especially promising candidates for non-invasive clinical diagnosis of breast cancer with easy adaptability to a broad range of settings. Due to the inherent miniaturization ability of electrochemical biosensors and their compatibility with standard microfabrication and semiconductor technologies, simple, accurate, portable, and inexpensive biosensing platforms have been developed for circulating breast cancer biomarkers at different molecular levels. However, a major limitation is the need for multiple steps and labeled reagents in the preparation of the biosensor before electrochemical detection takes place. While electrochemical detection often achieves the highest sensitivities due to the use of surface-bound probes and the preconcentration of the analyte on the sensor surface, single-step optical readout strategies—generally performed in homogeneous solution—are often less sensitive. In addition, electrochemical detection is extremely compatible with high multiplexing capability and both low- and high-density electrochemical sensor arrays can be conveniently fabricated by adopting the standard microfabrication technology. These breakthroughs and the superseding capabilities already demonstrated allow envisioning faster progress and greater applications of electrochemical compared to optical biosensing for non-invasive breast cancer diagnosis.

However, despite the encouraging capabilities demonstrated, all the electrochemical biosensors reported so far for the determination of circulating breast cancer biomarkers remain in the proof of concept or prototype stages of development, and none of them has been evaluated with a large enough number of real patient samples to assess their reliable clinical validation. Therefore, an exhaustive validation through the analysis of large numbers of real patient samples and the comparison against other available competitive methodologies remains to be addressed. The future translation into commercial products demands also the improvement of reproducibility in the preparation of different batches, the robustness against environmental variables by optimizing the best storage stability and transportation conditions to ensure appropriate functionality, and their simplicity of preparation and use. Additional efforts should be focused also on the biosensors’ integration into automated and miniaturized systems. It is worth mentioning also that, despite the significant progress made regarding the non-specific adsorption and multiplexed requirement issues, further efforts will be needed to detect a larger number of analytes in scarcely diluted liquid biopsies. 

Therefore, although the replacement of techniques currently used for the determination of circulating breast cancer biomarkers by electrochemical biosensing devices is clearly not imminent, they promise a paradigmatic shift in the way to conduct disease diagnosis and health monitoring in the near future, allowing for more rapid clinical decision-making, with the corresponding healthcare costs and patient stress reduction it represents. This shift will be facilitated by developments in molecular biology, nanofabrication methods and labeling, and likely will cause electrochemical biosensors to find an important niche for determining circulating breast cancer biomarkers in the hospital routine. 

In summary, although extremely sensitive and accurate clinical measurements of circulating biomarkers for early detection of breast cancer pose a formidable challenge, the wide range of possibilities and interesting capabilities described so far make feasible the future availability of inexpensive devices for reliable on-the-spot cancer diagnosis using electrochemical biosensors. These methodologies are envisaged to provide, apart from a reliable earlier diagnosis which will lead to improved therapeutic outcomes, also a better fundamental understanding of this disease progression.

## Figures and Tables

**Table 1:** Electrochemical genosensors for the determination of circulating breast cancer genetic biomarkers.

Electrode	Method	Target Oligonucleotide	Electrochemical Technique/Redox Probe	L.R.	LOD	Applicability	Ref.
Gold electrode	Immobilization of a binary self-assembled monolayer composed of a thiolated specific capture DNA probe (BRCA1 5382 insC mutation detection) and 6-mercapto-1-hexanol (MCH) on the gold electrode.	*BRCA1* gene mutations	EIS/[Fe(CN)_6_]^4−/3−^	1.0 × 10^−19^‒1.0 × 10^−7^ M	4.6 × 10^−20^ M	Genomic DNA extracted from peripheral blood samples	[19]
GCE	Preparation of an antifouling GCE by highly cross-linking of polyethylene glycol films containing amine groups where AuNPs were self-assembled for further immobilization of a BRCA1 related 19-mer DNA sequence.	*BRCA1* gene mutations	EIS/[Fe(CN)_6_]^4−/3−^	50.0 fM‒1.0 nM	1.72 fM	Doped serum samples	[2]
AuNPs-modified SPCE	Immobilization of a thiolated RNA probe, p19 binding onto the RNA-RNA duplex formed on the electrode surface by direct hybridization, and displacemen t of the attached p19 by incubation in a mixture of a target miRNA and a nonthiolated RNA probe at high concentration.	miRNA-21, miRNA-32, miRNA-122	SWV/K_3_[Fe(CN)_6_] and [Ru(NH_3_)_6_]Cl_3_	10 aM–1 μM	5 aM	Human serum samples	[22]
Au-SPE	Immobilization of a thiolated RNA probe and direct hybridization.	miRNA-155	SWV/[Fe(CN)_6_]^3−/4−^	10 aM–1.0 nM	5.7 aM	Human serum samples	[3]
GCE functionalized with AuNRs decorated on GO sheets	Immobilization of a thiolated RNA probe and direct hybridization.	miRNA-155	DPV/OB	2.0 fM–8.0 pM	0.6 fM	Spiked human plasma samples	[7]
Au-SPE	Immobilization of a thiolated capture probe on the gold electrode. In the presence of the target miRNA the stem-loop structure of such capture probe is unfolded and hybridizes with the DNA concatamers.	miRNA-21	DPV/[Ru(NH_3_)_6_]^3+^	100 aM–100 pM	100 aM	Human serum samples	[23]
GCE	Bi-functional Janus probe containing both the complementary RNA oligonucleotide sequence for the target miRNA and the DNA sequence used as primer for long-range self-assembled DNA concatamers. The RNA duplexes generated by hybridization between the Janus probe and the target miRNA was selectively captured onto the surface of p19-MBs and long DNA concatamers anchored to the MBs were regenerated by addition of two specific auxiliary probes.	miRNA-21	SWV/DSA intercalated	20 aM–100 aM	6 aM	Human serum samples	[24]
GCE modified with AuNPs using PDDA	Immobilization of a thiolated capture probe with MB labeled at 5′ end. Hybridization with the target miRNA in the presence of an auxiliary probe, forming a star trigon structure on the electrode surface. The endonuclease cleaves the capture probe on capture/auxiliary probes duplex, releasing microRNA and auxiliary probe back to the solution.	miRNA-21	SWV/MB of the AP	100 aM–1 nM	30 aM	Human serum samples	[6]
Gold electrode	Immobilization of a hairpin-like DNA probe modified with a thiol and a biotin on a gold electrode through the thiolated moiety. After hybridization with the target miRNA the biotin group in the capture probe was forced away from the electrode surface, allowing for the coupling of Strept-ALP.	miRNA-21	Amperometry/AA + FcM + TCEP	0.5 fM–1 pM	0.2 fM	Human serum samples	[25]
Gold electrode	Immobilization of a thiolated capture probe on the gold electrode. In the presence of miRNA-21, a sandwiched DNA complex was formed between the capture and a MB-labeled G-rich detection probe attached onto AuNPs. Upon addition of K^+^, the structure of the detector probe changed to a G-quadruplex and the iridium(III) complex could selectively interact with it and catalyze the reduction of H_2_O_2_, in the presence of MB.	miRNA-21	CV/H_2_O_2_ + MB	5.0 fM–1.0 pM	1.6 fM	Spiked human serum samples	[21]
Tungsten oxide (WO_3_)-graphene composites coupled with AuNPs	Immobilization of a thiol-terminated capture probe H1 immobilized on the electrode through Au–S interaction. Hybridization with the target miRNA opens H1 hairpin structure and another stable biotinylated hairpin DNA (H2) displaced target miRNA releasing it back to the sample solution for the next cycle. After this cyclic process a large amount of H1-H2 duplex was produced and a lot of Strept-ALP molecules were immobilized on the electrode.	miRNA-21	DPV/AA + FcM + TCEP	0.1 fM–100 pM	0.05 fM	Human serum samples	[20]
GCE	Target recycling, nicking-replication reaction and DNAzyme catalysis coupling.	miRNA-21	Amperometry/TMB^+^ H_2_O_2_	1 aM–100 pM	0.5 aM	Spiked human serum samples	[26]
MGCE	Method based on both the DSNATR and capture probes enriched from the solution to the electrode surface using MBs. In the absence of the target miRNA, the capture probes cannot be hydrolyzed due to the low activity of duplex-specific nuclease against ss-DNA, the intact capture probes could be attached to Strep-MBs and hence onto the surface of a magnetic-GCE, resulting in a compact negatively charged layer, giving rise to a large charge-transfer resistance measured in the presence of [Fe(CN)_6_]^4−/3−^. Conversely, in the presence of miRNA-21, it hybridized with the capture probes to form a DNA-RNA heteroduplex and the DSN hydrolyzed the target-binding part of the capture probe thus liberating the intact miRNA-21, which was able to trigger the permanent hydrolysis of multiple capture probes which were, finally, all digested. Therefore, the negatively charged layer could not be formed and a small charge-transfer resistance was measured.	miRNA-21	EIS/[Fe(CN)_6_]^4−/3−^	0.5–40 fM	60 aM	Human serum samples	[27]
Gold electrode	The molecular beacon template consisted of three domains: a miRNA-binding domain, a recognition domain by Nb.BbvCI, and an amplification domain for producing DNA triggers. In the presence of target miRNA, the specific hybridization with the corresponding domain opened the hairpin structure of the molecular beacon template, which led to a part duplex. Then, the target miRNA was extended along the template to form a complete duplex by Klenow fragment and dNTPs. Subsequently, the nicking enzyme specifically recognized the duplex nicking site, cleaving the upper extended DNA strand and exposing a new replication site for polymerase. While one part of DNA triggers bound to the capture probes immobilized on the gold electrode the other part hybridized with the biotinylated detector probe, which could be linked to Strep-ALP.	miRNA-222	DPV/α-NP	50 pM–10 nM	40 pM	Spiked serum samples	[28]

Abbreviations: AuNPs: gold nanoparticles; AuNRs: gold nanorods; CV: cyclic voltammetry; DSA: 5,7-dinitro-2-sulfo-acridone; DPV: differential pulse voltammetry; DSNATR: duplex-specific nuclease assisted target recycling; ECC: electrochemical-chemical-chemical; EIS: electrochemical impedance spectroscopy; FcM: ferrocenemethanol; GCE: glassy carbon electrode; GO: graphene oxide; α-NP: α-naphthyl phosphate; MB: methylene blue; MGCE: magnetic glassy carbon electrode; OB: Oracet Blue; PDDA: diallyldimethylammonium chloride; Strept-ALP: streptavidin-conjugated alkaline phosphatase; SWV: square wave voltammetry; TCEP: tris(2-carboxyethyl)phosphine; TMB: 3,3´,5,5´ tetramethylbenzidine.
